# Chromosomal Instability Characterizes Pediatric Medulloblastoma but Is Not Tolerated in the Developing Cerebellum

**DOI:** 10.3390/ijms23179852

**Published:** 2022-08-30

**Authors:** Irena Bočkaj, Tosca E. I. Martini, Marlinde J. Smit, Inna Armandari, Bjorn Bakker, René Wardenaar, Tiny G. J. Meeuwsen-de Boer, Petra L. Bakker, Diana C. J. Spierings, Eelco W. Hoving, Victor Guryev, Floris Foijer, Sophia W. M. Bruggeman

**Affiliations:** 1 European Research Institute for the Biology of Ageing (ERIBA), University Medical Center Groningen, University of Groningen, 9713 AV Groningen, The Netherlands; 2 Department of Pathology and Medical Biology, University Medical Center Groningen, University of Groningen, 9700 RB Groningen, The Netherlands; 3 Princess Máxima Center for Pediatric Oncology, 3584 EA Utrecht, The Netherlands

**Keywords:** medulloblastoma, genomic instability, chromosomal instability, aneuploidy, genotoxic stress, cerebellar granule neuron progenitors, cerebellar development

## Abstract

Medulloblastoma is a pediatric brain malignancy that consists of four transcriptional subgroups. Structural and numerical aneuploidy are common in all subgroups, although they are particularly profound in Group 3 and Group 4 medulloblastoma and in a subtype of SHH medulloblastoma termed SHHα. This suggests that chromosomal instability (CIN), the process leading to aneuploidy, is an important player in medulloblastoma pathophysiology. However, it is not known if there is ongoing CIN in medulloblastoma or if CIN affects the developing cerebellum and promotes tumor formation. To investigate this, we performed karyotyping of single medulloblastoma cells and demonstrated the presence of distinct tumor cell clones harboring unique copy number alterations, which is suggestive of ongoing CIN. We also found enrichment for processes related to DNA replication, repair, and mitosis in both SHH medulloblastoma and in the highly proliferative compartment of the presumed tumor cell lineage-of-origin, the latter also being sensitive to genotoxic stress. However, when challenging these tumor cells-of-origin with genetic lesions inducing CIN using transgenic mouse modeling, we found no evidence for large chromosomal aberrations in the cerebellum or for medulloblastoma formation. We therefore conclude that without a background of specific genetic mutations, CIN is not tolerated in the developing cerebellum in vivo and, thus, by itself is not sufficient to initiate medulloblastoma.

## 1. Introduction

Genomic instability (GIN) can be defined as a high frequency of genetic alterations, ranging from single nucleotide alterations to whole chromosome aneuploidies. Since many human cancers are prone to GIN and GIN drives genome evolution, it is considered one of the enabling hallmarks of cancer [1]. Interestingly, pediatric malignancies typically exhibit a low burden of chromosomal abnormalities and mutations, yet there are some pediatric brain cancers that are highly genomically instable [2]. These include medulloblastoma, the most common pediatric brain malignancy [3,4].

Medulloblastoma is a tumor that arises from the cerebellum. It consists of four subgroups, each thought to have a separate etiology and molecular driving force: Sonic Hedgehog (SHH), WNT, Group 3 and Group 4 [5,6,7]. Structural and numerical aneuploidy are common features among all subgroups, although they are particularly profound in Group 3, Group 4, and a subtype of SHH medulloblastoma termed SHHα [2,6,7,8,9]. The latter subtype has a particularly unfavorable outcome when associated with *TP53* mutations [6]. Interestingly, *TP53* mutant SHH medulloblastoma exhibits an increased structural variant load and typical losses of 9q, 10q, and 17p [2,6,10,11]. These recurrent arm-sized copy number variations suggest that genomic instability caused by chromosomal instability (CIN) may be involved in medulloblastoma [9]. 

However, the question as to whether there is actual ongoing CIN in medulloblastoma has not yet been fully addressed, nor is it known if CIN occurs in the developing cerebellum and can act as a driving event in medulloblastoma. To answer these questions, it is important to understand the developmental processes that surround tumor initiation. The presumed cell-of-origin for SHH and Group 3 medulloblastoma is part of the cerebellar granule neuron progenitor (CGNP) cell lineage [12,13,14,15,16,17,18,19]. CGNPs are born in early neural development but remain present for almost two years after birth in humans, and up to three weeks in mice [20,21,22,23,24]. Murine CGNPs are specified in the upper rhombic lip, from where they form a secondary germinal zone (external granular layer or EGL) on the cerebellar surface that exhibits a peak in proliferation around birth [20,21,22,23]. Towards the end of cerebellar development, differentiating granule neurons cease proliferation and form the internal granular layer (IGL) of the cerebellum [22,25,26,27,28]. It is tempting to speculate that this perinatal surge in proliferation brings along a risk of genomic instability, as these cells might not have sufficient time to repair replication stress-induced DNA damage. 

In this study, we addressed whether genomic instability, and in particular CIN, play a role in medulloblastoma and cerebellar development. Using single cell karyotype analysis, we identified different subclones with unique copy number alterations in each tested medulloblastoma biopsy, suggesting that there is indeed ongoing CIN in human medulloblastoma. We also found that normal perinatal murine CGNPs show enrichment for processes related to DNA replication, DNA repair, and mitotic progression that are also enriched in a subset of SHH medulloblastoma patients [18]. We further established that primary neonatal CGNPs are sensitive to the CIN-provoking Mps1 inhibitor reversine in vitro [29], and that there is age-dependent sensitivity to ionizing radiation in these cells. However, when neonatal CGNPs are challenged in vivo with genetic lesions inducing CIN, we found no evidence for the occurrence of large chromosomal aberrations or medulloblastoma formation [13,16,30,31,32]. Together, these findings suggest that whereas perinatal CGNPs are differentially sensitive to genotoxic stress, CIN is not tolerated in the developing cerebellum in vivo and therefore by itself is not sufficient to initiate medulloblastoma.

## 2. Results

### 2.1. Single Cell Karyotyping Reveals Genomic Instability and Intra-Tumor Heterogeneity in Pediatric Medulloblastoma, Indicative of Ongoing CIN

**Figure 1:**
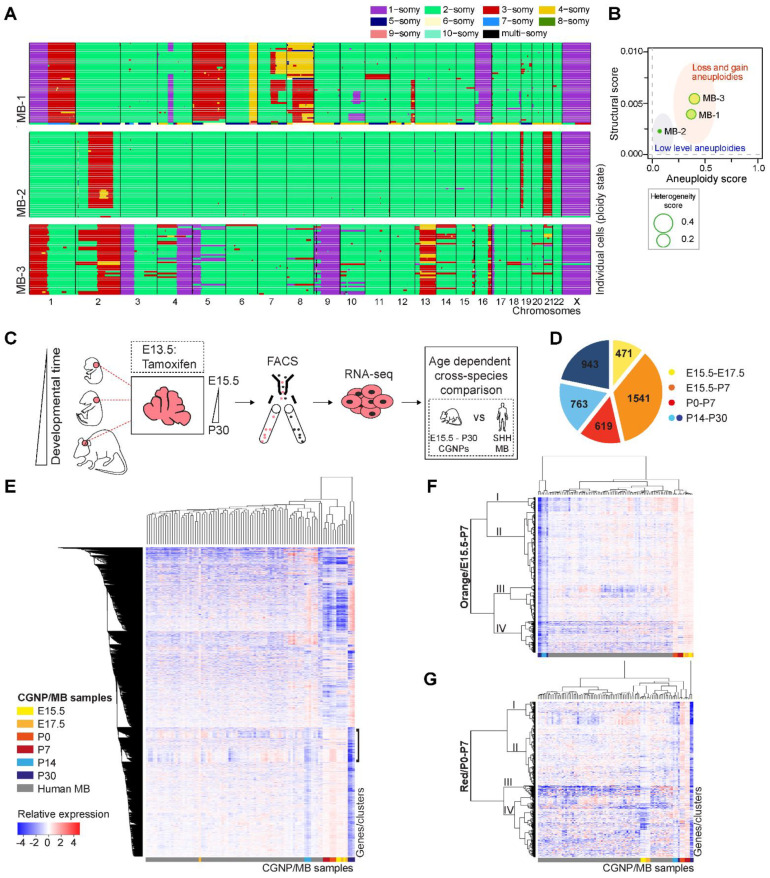
Single cell whole genome sequencing (scWGS) reveals genomic instability and intra-tumor heterogeneity in pediatric medulloblastoma. (**A**) scWGS data of three human medulloblastoma samples. Each colored line represents the karyotype of a single cell (MB-1, n = 44; MB-2, n = 46; MB-3, n = 38 cells). Chromosomes are indicated on the *x*-axis. Colors indicate the ploidy state of their corresponding region, as stated in the legend. (**B**) Structure, aneuploidy and heterogeneity plot of n = 3 pediatric medulloblastomas. The structural score (plotted on the *Y*-axis) represents the number of structural abnormalities observed in the tumor, as it quantifies the frequency of copy number changes within chromosomes. The aneuploidy score (plotted on the *X*-axis) quantifies the frequency of large copy number changes (whole chromosome or chromosome arm gains or losses). The heterogeneity score, represented by circle size, measures the extent of structural and numerical variation between the cells from one tumor. (**C**) Schematic overview of the setup of the transcriptome analysis study. Transgenic mice allowing spatiotemporally controlled labelling of cerebellar granule neuron progenitors (CGNPs) with tdTomato were treated with Tamoxifen on embryonic day E13.5 of pregnancy. tdTomato+ cerebella were harvested between embryonic day E15 and postnatal day P30. tdTomato+ CGNPs were sorted using FACS and subjected to transcriptome analysis and subsequent cross-species analyses with a cohort of human SHH medulloblastoma. Adapted with permission from [18]. (**D**) Pie chart summarizing five major groups of differentially expressed genes as a function of CGNP developmental age. Yellow group (E15.5-E17.5, n = 471 genes); orange group (E15.5-P7, n = 1541 genes); red group (P0-P7, n = 619 genes); light blue group (P14-P30, n = 763); and dark blue group (P14-P30, n = 943). Reproduced with permission from [18]. (**E**) Heatmap showing a cross-species unsupervised hierarchical clustering analysis of human SHH medulloblastoma genes and CGNP orthologous genes (CGNP genes as indicated in (**D**)). Medulloblastoma and CGNP samples are plotted on the *X*-axis. Genes are plotted on the *Y*-axis. The gene cluster showing similarities in gene expression patterns between SHH medulloblastoma and CGNP samples is indicated by the black bracket. (**F**) Heatmap showing a cross-species unsupervised hierarchical clustering analysis of human SHH medulloblastoma genes and CGNP orthologous genes of the orange/E15-P7 gene group (CGNP genes as indicated in (**D**)). Medulloblastoma and CGNP samples are plotted on the *X*-axis. Genes are plotted on the *Y*-axis. (**G**) Heatmap showing a cross-species unsupervised hierarchical clustering analysis of human SHH medulloblastoma genes and CGNP orthologous genes of the red/P0-P7 gene group (CGNP genes as indicated in (**D**)). Medulloblastoma and CGNP samples are plotted on the *X*-axis. Genes are plotted on the *Y*-axis.

To investigate if ongoing genomic instability, and in particular CIN, is a feature of pediatric medulloblastoma, DNA single cell sequencing technology was employed (e.g., shallow single cell Whole Genome Sequencing or scWGS). This approach allows for the assessment of larger copy number variations at the single cell level and, thereby, karyotype clonality and heterogeneity within a tumor [33,34]. When we looked at the karyotypes of single cells isolated from three untreated medulloblastoma samples, we found large differences between tumor samples, with MB-1 and MB-3 being the most heterogeneous and showing the greatest divergence from euploidy (Figure 1A,B). Furthermore, most profoundly in MB-1 and MB-3, we observed coexisting clones, as evidenced by single cells with varying chromosome compositions, indicating intratumor heterogeneity and, thus, ongoing CIN (Figure 1A,B). We then wanted to perform live imaging on cultured primary medulloblastoma cells to confirm that there is indeed ongoing CIN in these cells. However, we observed that already during a relatively short period of cell culturing, there was a significant loss of karyotype diversity, indicating strong clonal selection in vitro (data not shown). This prompted us to further study genomic and chromosomal instability using mouse modeling.

### 2.2. Enrichment for DNA Replication, Repair, and Mitosis-Related Processes Is Dependent on Developmental Age in CGNPs and Characterizes a Subset of Medulloblastoma Patients

In a recent study, we performed a transcriptome analysis on CGNPs isolated from mouse developing cerebellum at different time points ranging from embryonic day 15 (E15.5) to postnatal day 30 (P30) (Figure 1C) [18]. We reported that developing murine CGNPs partially resemble human SHH medulloblastoma, in line with the CGNP lineage being the cell-of-origin for this tumor. We further found that CGNPs exhibit unique transcriptional profiles as a function of developmental age, suggesting that the identity of the CGNP population changes over time (Figure 1D) [18]. For instance, in CGNPs aged E15.5-E17.5, there are n = 471 unique genes highly expressed (yellow gene group), whereas in CGNPs aged P0-P7 n = 619 genes are highly expressed (red gene group) (Figure 1D). Interestingly, we found an enrichment of biological processes related to cell cycle transition and DNA repair in early and perinatal CGNPs (E15.5-P7, orange and red gene groups) [18]. This fits with the hypothesis that the perinatal surge in CGNP proliferation could sensitize these cells to oncogenic transformation.

For the present study, we selected all murine CGNP genes that were differentially expressed in time as identified earlier by us (i.e., all genes of the gene groups of Figure 1D) [18], and performed cross-species unsupervised hierarchical clustering with a cohort of human SHH medulloblastoma transcriptomes (note that SHH medulloblastoma gene expression data were extracted from a publicly available database of medulloblastoma transcriptomes) (Figure 1C–E) [35]. We identified a cluster of genes (Figure 1E, indicated by the black bracket) that showed similar expression patterns in murine CGNPs and patients. Curiously, a part of the patients’ gene expression resembled expression patterns found in young CGNPs (E15.5-P7), whereas another subset of patients exhibited a reciprocal pattern and, therefore, appeared more similar to older CGNPs (P30). To investigate if these genes belonged to specific age groups of CGNPs (e.g., to the yellow (E15.5-E17.5); orange (E15.5-P7); red (P0-P7); or light and dark blue gene groups (P14-P30) shown in Figure 1D), we repeated the cross-species clustering experiment using only the genes specifically enriched in one CGNP age group at a time, yielding a total of five clustering ana-lyses (Figure 1F,G and Figure S1A–C). We did not find gene expression patterns that were clearly similar between SHH medulloblastoma and CGNPs in the yellow and blue gene groups, indicating that there are not many similarities in gene expression between human SHH medulloblastoma and either the youngest (yellow) or oldest (blue) CGNPs (Figure S1A–C). However, in the early to perinatal gene groups (i.e., the orange group (Figure 1F) and the red group (Figure 1G)), there were several gene subclusters (identified by the branching tree, indicated with Roman numerals) that were similarly expressed in CGNPs and the medulloblastoma samples.

**Figure 2:**
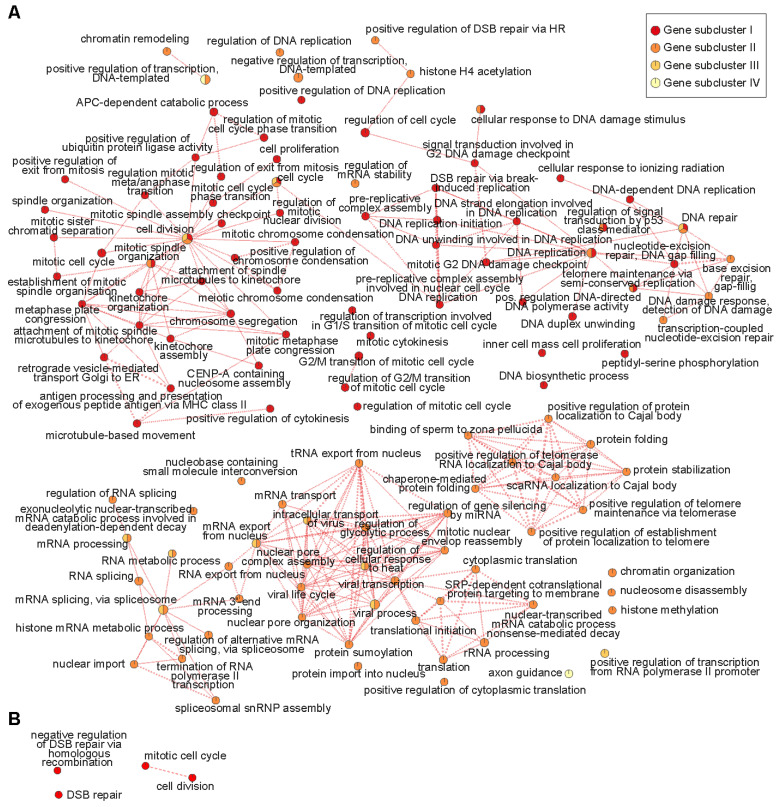
Enrichment for DNA replication, repair, and mitosis-related processes in gene subclusters that show comparable gene expression patterns between medulloblastoma patients and E15-P7 CGNPs. (**A**) Gene ontological analysis showing enriched biological processes in gene subclusters I-IV that exhibit comparable gene expression patterns in E15-P7 CGNPs (orange group) and SHH medulloblastomas (see also Figure 1D,F). Each node represents a biological process. Biological processes connected by edges have genes in common. Enriched biological processes were determined with the Database of Annotation, Visualization and Integrated Discovery (DAVID), v.6.8 (Benjamini-corrected q = 0.1, *p* = 0.01) and visualized with the Enrichment Map app in Cytoscape. Red nodes: enriched in gene subcluster I of Figure 1F; dark orange nodes: enriched in gene subcluster II; light orange nodes: enriched in gene subcluster III; yellow nodes: enriched in gene subcluster IV. (**B**) Gene ontological analysis showing enriched biological processes in gene subclusters I-IV that exhibit compared gene expression patterns in P0-P7 CGNPs (red group) and SHH medulloblastomas (see also Figure 1D,G).

This finding prompted us to explore potential biological mechanisms underlying these similarities in expression patterns. To this end, we identified enriched biological processes (Gene Ontology) within the gene subclusters using the Database for Annotation, Visualization and Integrated Discovery (DAVID), and visualized the results using Cytoscape (Figure 2A corresponding to Figure 1F/orange gene group and Figure 2B corresponding to Figure 1G/red gene group) [36]. Here, enriched biological processes can be defined as Gene Ontology-based biological processes, for which more genes from the subcluster gene sets are being called, as would be expected from a random gene set. Hence, an identified enriched biological process may play a role in the corresponding sample(s). 

We found that especially subclusters I and II of the orange group (E15.5-P7, Figure 2A) contained multiple enriched biological processes. Intriguingly, many of them are related to RNA processing (for example: mRNA splicing via spliceosome; termination of RNA polymerase II transcription); DNA replication (for example: DNA replication; DNA replication initiation); DNA repair (for example: nucleotide-excision repair-DNA gap filling; DNA damage response-detection of DNA damage); and mitotic cell division (for example: mitotic spindle organization; chromosome segregation). Together, these findings suggest that these processes, which we already knew to be particularly active in perinatal CGNPs and coincide with the Sonic Hedgehog-induced peak in CGNP proliferation that takes place around birth, are also implicated in SHH medulloblastoma [20,21,22,23].

### 2.3. Neonatal CGNPs Are Sensitive to Ionizing Radiation and CIN Induction

**Figure 3:**
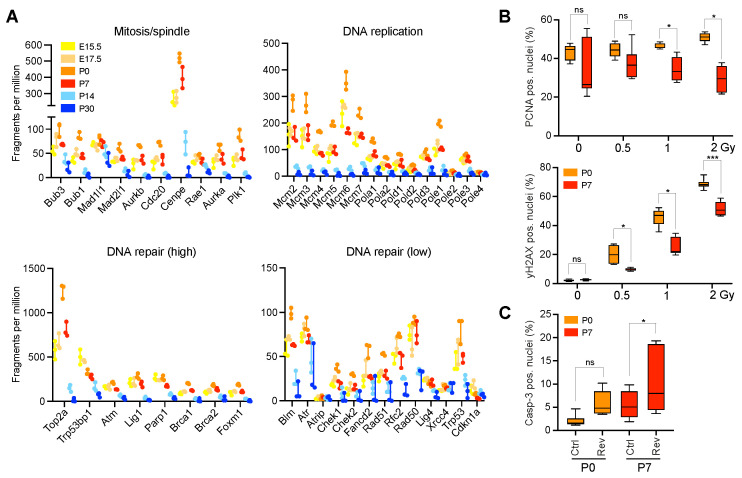
Early postnatal CGNPs are sensitive to DNA damage and CIN-inducing stressors. (**A**) Plots showing relative gene expression levels (in fragments per million) of individual genes involved in mitosis/spindle (upper left panel), DNA replication (upper right panel), and DNA repair (relatively high expressed genes: lower left panel, relatively low expressed genes: lower right panel) in CGNPs isolated between embryonic day E15.5 and postnatal day P30. Results represent range and individual data points. n = 3 samples per developmental time point. (**B**) Box and whisker plots showing the fraction of PCNA positive (upper panel) or γH2AX positive (lower panel) nuclei in Y-ray irradiated primary P0 (orange bars) and P7 (red bars) CGNP cultures. Radiation dose is plotted on the *X*-axis (Gy = Grey). Whiskers represent min to max. n = 6 biological replicates are plotted per condition. *p* values were determined using multiple *t*-tests with Holm-Sidak correction. * *p* < 0.05, ns = not significant. (**C**) Box and whisker plot showing the fraction of Caspase-3 positive nuclei in primary P0 (orange bars) and P7 (red bars) CGNP cultures treated with 250 nM of reversine or DMSO (Ctrl). Whiskers represent min to max. At least n = 8 biological replicated are plotted per condition. *p* values were determined using an ordinary one-way ANOVA with Sidak correction. * *p* < 0.05, *** *p* < 0.001, ns = not significant.

We then analyzed the expression of individual genes involved in DNA replication, mitosis, and DNA repair in developing CGNPs (Figure 3A). This confirmed the gene ontology analysis, as we typically observed the highest gene expression between E17.5 and P7. To test the hypothesis that proliferating, neonatal CGNPs are indeed sensitive to DNA damage and CIN, we isolated primary CGNPs from P0 and P7 murine cerebella and subjected them to Y-ray irradiation or the CIN-inducing Mps1 inhibitor reversine (Figure 3B,C) [29]. We found no significant difference in proliferation between control and low dose-irradiated P0 and P7 CGNPs (Figure 3B, upper panel). Interestingly, while irradiated CGNP cultures exhibited an increased expression of γH2AX already at low dose irradiation, indicating that they are indeed sensitive to the induction of DNA double strand breaks (DSBs) at this time, the effect was stronger in P0 cells compared to P7 (Figure 3B, lower panel). We further observed that upon treatment with reversine, apoptosis increased in both P0 and P7 cultures (Figure 3C). Thus, perinatal CGNPs are sensitive to genotoxic stress, the extent of which can be influenced by developmental age.

### 2.4. Cerebellum-Specific Deletion of Mitotic Checkpoint Protein Mad2l1 and Tumor Suppressor Trp53 in Neonatal Mice Does Not Lead to Medulloblastoma Formation

To investigate the impact of CIN and aneuploidy on cells and tissues in vivo, a number of mouse models have been established previously that rely on the tissue-specific deletion of mitotic checkpoint or kinetochore components to circumvent systemic toxicity [37]. For this study, we wanted to address if CIN and the resulting aneuploidy influence the developing cerebellum and cause tumor formation. To this end, we generated a transgenic mouse model that allows the spatiotemporally controlled deletion of mitotic spindle checkpoint component *Mad2l1* from developing CGNPs using floxed *Mad2l1* alleles and the Math1CreER^T2^-tdTomato driver/reporter [18,23,38,39,40,41,42]. To mitigate potential deleterious effects on CGNP viability and promote tumor formation, we also included the conditional deletion of floxed *Trp53*, a tumor suppressor gene that is frequently mutated in medulloblastoma [6,40]. 

**Figure 4:**
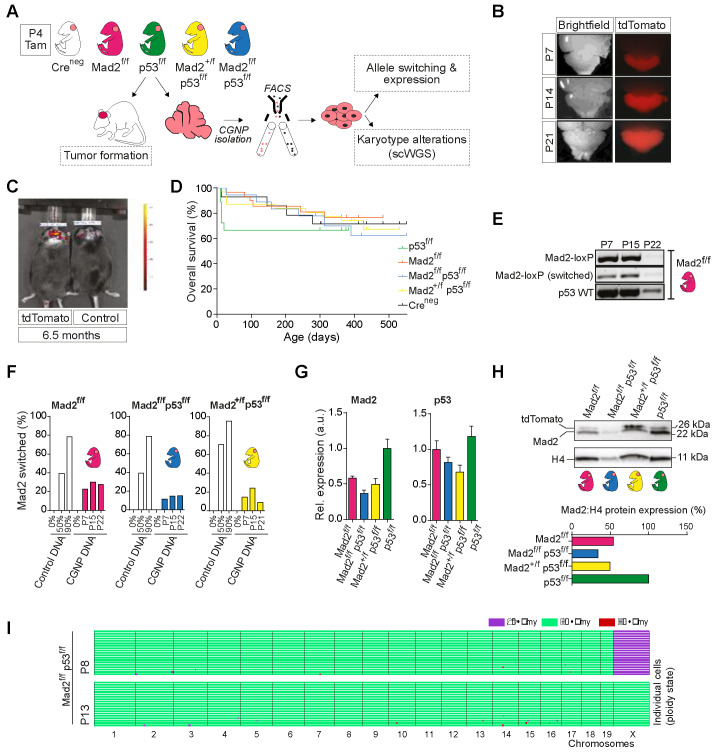
Induction of CIN/aneuploidy is not tolerated in the developing cerebellum and does not provoke medulloblastoma. (**A**) Schematic overview of the transgenic mouse model and experimental setup. Five experimental groups of P4 pups containing the tdTomato reporter were generated: Cre^Negative^ (white), Mad2^f/f^ (pink), p53^f/f^ (green); Mad2^+/f^, p53^f/f^ (yellow), and Mad2^f/f^; p53^f/f^ (blue). P4 pups were treated once with Tamoxifen by oral gavaging. Mice were monitored for tumor formation. Additionally, fluorescent CGNPs were isolated at different time points for further analyses. (**B**) Stereoscopic fluorescent images showing tdTomato expression in cerebellum between postnatal day P7 and P21. (**C**) IVIS images demonstrating tdTomato expression in the cerebellum of a 6.5-month-old transgenic and control mouse. (**D**) Kaplan–Meier survival curve showing overall survival of the five experimental groups described at (**A**). (**E**) Conventional genomic PCR assessing the switching efficiency of the floxed Mad2l1 alleles in Mad2^f/f^ cerebella of postnatal day P7 (n = 1), P15 (n = 1), and P22 (n = 1) mice. Upper bands: floxed (unswitched) allele; middle bands: switched Mad2l1 allele; lower bands: wild type Trp53. (**F**) Bar charts showing the switching efficiency of the Mad2l1 floxed allele in Mad2^f/f^ (n = 1), Mad2^f/f^; p53^f/f^ (n = 1), and Mad2^+/f^; p53^f/f^ (n = 1) sorted CGNPs or titrated control at P7, P15, and P21 at the genomic level, as assessed by quantitative genomic PCR. (**G**) Bar charts showing the switching efficiency of the Mad2l1 and Trp53 floxed alleles at the mRNA level in P7 CGNPs, as assessed by quantitative RT-PCR. (**H**) Western blot showing the switching efficiency of the Mad2l1 floxed allele at the protein level in P7 CGNPs. (**I**) scWGS data of sorted P8 and P13 Mad2^f/f^; p53^f/f^ CGNPs. Each colored line represents the karyotype of a single cell (P8, n = 19; P13, n = 19 cells). Chromosomes are indicated on the *X*-axis. Colors indicate the ploidy state of their corresponding region, as stated in the legend.

From the CGNP transcriptome data, we confirmed that both the *Mad2l1* and *Trp53* genes are highly expressed in neonatal CGNPs (Figure 3A). We then generated five groups of experimental animals: a MathCreER^T2^-driver/reporter-only group (hereafter referred to as: Cre^Negative^ (white)); a Mad2l1-only, and a Trp53-only deletion group (referred to as Mad2^f/f^ (pink), or p53^f/f^ (green)); and two compound deletion mutant groups that were expected to exhibit mild and severe CIN, respectively (Mad2^+/f^; p53^f/f^ (yellow); and Mad2^f/f^; p53^f/f^ (blue)) (Figure 4A). 

Deletion of the conditional alleles was induced following the treatment of P4 pups with the drug Tamoxifen. As the transgenic mouse model contained a floxed tdTomato reporter that is expressed upon the successful activity of the Cre recombinase, we were able to confirm that cells targeted by Cre persisted in the cerebellum up to at least 6 months of age (Figure 4B,C). However, although we monitored the different experimental groups for more than 1.5 years, none of them developed medulloblastoma (Figure 4D), nor did we find any neurodevelopmental abnormalities (data not shown).

To exclude the possibility of less efficient switching of the *Mad2l1* and *Trp53* floxed alleles compared to the tdTomato reporter, we first assessed *Mad2l1* switching in the Mad2^f/f^ group by a conventional PCR (Figure 4E). This showed clear evidence of the expected recombination at the *Mad2l1* locus in P7, P15, and P22 cerebellum (Figure 4E, middle panel), but also a clear product for the unrecombined *Mad2l1* allele, indicating that Cre-mediated recombination was partial. A qPCR further confirmed partial *Mad2l1* switching in all three Mad2^f/f^ groups at comparable levels (Figure 4F). This was further validated by a MAD2 protein and mRNA expression analysis (Figure 4G,H). Surprisingly, whereas developing CGNPs seem to tolerate the (partial) loss of *Mad2l1*, switching of the *Trp53* conditional allele was less efficient, indicating selection against this (Figure 4G and Figure S2A).

### 2.5. The Developing Cerebellum Does Not Appear to Tolerate Aneuploidy

Given that no tumors or developmental abnormalities were found, despite the reduced expression of Mad2l1, we investigated if recently switched Mad2l1^f/f^ CGNPs exhibited evidence of CIN or aneuploidy at the single cell level. We sorted tdTomato^+^ CGNPs from P8 and P13 Mad2^f/f^; p53^f/f^ cerebella and isolated the G0/G1 population for scWGS karyotype analysis (Figure S2B). Surprisingly, no aneuploidies or copy number alterations were detected in any of the sequenced cells (Figure 4I). This suggests that chromosomal instability is not tolerated in vivo in the developing cerebellum and explains the absence of medulloblastoma formation in our mouse model.

## 3. Discussion

Although aneuploidy is a prominent feature of pediatric SHH medulloblastoma, it remains unclear if chromosomal instability plays a role in driving this cancer. In this study, we showed that aneuploid pediatric medulloblastoma cells are heterogenous in their karyotype composition, suggesting that ongoing chromosomal rearrangements are indeed a characteristic of human medulloblastoma, in line with recent findings [43]. Pa-radoxically, we also found that CIN and aneuploidy are selected against in normal progenitor cells of the developing cerebellum in vivo. These observations are in agreement with other studies reporting that, whereas cancer cells can survive with high rates of chromosomal mis-segregation and aneuploidy, aneuploidy is detrimental for most normal cells [44]. This apparent paradox can be reconciled by the existence of a protective mechanism that safeguards CGNPs from collecting aneuploidies and other types of genomic damage. To allow tumor formation, this protective mechanism needs to be overcome.

### 3.1. Tolerance of CIN and Aneuploidy in the Developing Cerebellum

Why would such a protective mechanism exist? The answer to this question might be related to the fact that mature neurons are extremely long-lived—maintaining genomic integrity may, therefore, be crucial. DNA damage-induced genomic instability has previously been shown to naturally occur during neurogenesis in the cerebellum, and the fast proliferating perinatal CGNPs may be particularly vulnerable [45]. The enrichment for processes related to DNA replication, repair, and mitosis in neonatal CGNPs, together with the observed sensitivity to genotoxic stressors, support this idea. Endogenous DNA damage secondary to high replication rates and transcriptional activity creates a constant need to maintain genomic integrity and, therefore, adapt the DNA damage response [45]. The latter could mean that there is a preference for DNA damage-induced apoptosis rather than repair: emphasis is placed on maintaining genome integrity over accepting the risk of progenitor expansion with unrepaired DNA [46]. Thus, we propose that the response to *Mad2l1* loss-driven CIN triggers an apoptotic response that is part of the protective mechanism. Newly born aneuploid cells might be cleared right away and were, therefore, missed in our single cell sequencing experiments. The high turnover in the progenitor population will subsequently compensate for the loss of apoptotic aneuploid cells, explaining the absence of a developmental or tumor phenotype. So, altogether, the cerebellar compartment can be defined as intolerant to aneuploidy.

### 3.2. Overcoming Intolerance to CIN and Medulloblastoma Formation

To overcome this intolerance and allow tumor development, it is conceivable that additional genetic lesions are required that disrupt the protective mechanism. Surprisingly, the simultaneous loss of *Trp53* together with *Mad2l1* was not sufficient in our mouse model. In fact, there even appeared to be selection against this event. This was somewhat unexpected, since *Trp53* null cerebellum mouse models have been generated previously without obvious problems [47,48,49]. An explanation could be that in the cerebellum, the timing of *Trp53* deletion is crucial. Constitutive, early *Trp53* loss may be compatible with cerebellar development, as compensatory mechanisms may take over p53 function, whereas acute loss is not tolerated at critical, later phases in CGNP development. Alternatively, the mechanisms involved in the clearance of aneuploid cells are still functional in *Trp53* null cells.

Other additional genetic lesions that can overcome intolerance to CIN likely target the remaining protective checkpoints. They could, for instance, entail an overactivation of the SHH pathway to further stimulate hyperproliferation, or genes related to apoptosis. The possibility that the acquisition of a CIN phenotype is not beneficial early on in medulloblastoma formation, but rather facilitates tumor progression once sufficient genetic alterations have occurred to counteract the detrimental effects of CIN, should also be considered [50]. Hence, it is recommended to further explore these ideas in future studies, as understanding of the processes facilitating tumorigenesis will likely yield new therapeutic strategies.

## 4. Materials and Methods

### 4.1. Experimental Animals and Treatments

The Math1-CreERT2 tdTomato compound transgenic mouse model used for the RNA-seq analysis has been described previously [18]. In short, it was derived from the Math1CreERT2 [23] and Ai14 mouse strains [41] (The Jackson Laboratory, strains #007684 and #007914, Bar Harbor, ME, USA), and was in a C57BL6/mixed background. Timed mating was performed overnight and the following morning was considered E0.5. Pregnancies were detected by an increase in body weight at E13.5, after which the pregnant females received a single dose of Tamoxifen (2 mg/100 μL peanut oil, Sigma, St Louis, MO, USA) by oral gavage. 

To study CIN and aneuploidy in the developing cerebellum, two additional mouse strains were bred into the above-described model: the conditional *Mad2l1* deletion mouse strain [38,39,51], and the conditional *Trp53* deletion mouse strain [40]. Four days after birth (P4), the neonates received 0.6 mg of Tamoxifen in a total volume of 30 microliters dissolved in peanut oil by oral gavage. For oral gavage, a 24-gauge reusable gavage needle was used, 25 mm in length, with a round, blunted end (1.25 mm diameter) (Fine Science tools, 18061-24, Heidelberg, Germany). To monitor tumor development, mice were briefly sedated with isoflurane and imaged using an IVIS imager. The Kaplan–Meier method was used to determine overall survival.

Adult mice were sacrificed by asphyxiation (CO2), and neonatal mice until the age of P7 were killed by decapitation. Offspring from different gender were randomly assigned. The mice were conventionally housed, fed *ad libitum*, and routinely genotyped by PCR. All animal experiments were approved by the Institutional Animal Care and Use Committee of the University Medical Center Groningen, the Netherlands.

### 4.2. Hierarchical Clustering Analysis and Gene Ontology

For the comparison of gene expression profiles between mouse CGNP and human medulloblastoma patients, a published human medulloblastoma data set was used (GEO, accession number GSE49243). For CGNP gene expression, a data set previously published by our group was used (EBI array express, accession number E-MTAB-7399) [18]. Expression values were transformed into log2 fold change (compared to average expression of a gene across all patients). Unambiguous orthologs (one to one orthology) were determined using the Ensembl Biomart tool (http://www.ensembl.org/biomart, accessed on 1 February 2017). Unsupervised hierarchical clustering and heatmap plotting was performed using the gplots library. The Euclidean distance and average clustering method was used for analyzing all CGNP genes, and the Manhattan distance and Ward.D clustering method was used for analyzing genes per CGNP gene group.

A subsequent pathway enrichment analysis was performed, as described previously [18]. In short, the genes of the gene subclusters were uploaded to the Database for Annotation, Visualization and Integrated Discovery v6.8 (DAVID), and a subsequent analysis for the gene ontology of biological processes was performed [52]. Lists of enriched biological processes were imported into and visualized with the Enrichment Map app in Cytoscape v3.2.1 [53,54]. Benjamini-correction was performed with a moderately permissive q value of <0.1 and a *p*-value of <0.01. Enriched biological processes are defined as biological processes (Gene Ontology) for which more (sub)cluster genes are called than would be expected from a random gene set. 

For assessing individual CGNP gene expression, selected genes were extracted from the CGNP RNA-seq data set, normalized to fragments per million, and plotted using Prism 9.1.2 [18].

### 4.3. Isolation, Culturing and Sorting of Cerebellar Granule Neuron Progenitors

CGNPs for cell culture and cell sorting were harvested from transgenic cerebella, as described previously [18]. In short, cerebella were dissected and meninges were removed. A single cell suspension was prepared with a papain dissociation kit, according to the manufacturer’s instructions (Worthington, Lakewood, NJ, USA). 

For cell culturing, unsorted P0 and P7 CGNPs were resuspended in culture media (DMEM-F12 (Gibco, New York, NY, USA) supplemented with 1% N2 (Invitrogen, Waltham, MS, USA); 1.5% glucose (Invitrogen); 5 μM HEPES (Invitrogen); and 0.25 μg/mL SHH (R&D Systems, Minneapolis, MN, USA)). The cells were seeded on pre-coated plates with poly-D-lysine (100 μg/mL, Sigma) at a density of 250.000 cells per well in 24-well plates. For ionizing radiation experiments, the cells were irradiated the next morning with 0.5, 1 or 2 Gray of Y-rays from a ^137^Cesium source (IBL 637 Cesium-137 Y-ray-machine). The cells were fixed at 2 h post irradiation. For reversine treatment, the cells were treated with DMSO or 250 nM of reversine (Sigma) for 48 h and subsequently fixed. 

For gDNA, RNA, and protein isolations, tdTomato positive/DAPI negative cells were sorted on a Beckman Coulter MoFlo Astrios sorter (Brea, CA, USA). Cell pellets were stored at −80 °C until further processing. For the isolation of nuclei for scWGS, see below.

### 4.4. Immunofluorescence

Cultured CGNPs were fixed with 100% methanol (room temperature) and blocked in a solution containing 0.1% Triton-X-100 (Sigma); 1% bovine serum albumin (BSA, Sigma); and 0.05% Tween20 (Sigma) in PBS. Primary antibodies were: PCNA (1:1000, ab29, Abcam, Cambridge, UK); γH2AX (1:400, 9718, Cell Signaling, Beverly, MA, USA); and cleaved Caspase-3 (1:400, 9661, Cell Signaling). Secondary antibodies were Alexa Fluor 488 (1:500) and Alexa Fluor 568 (1:500, Invitrogen). The cells were counterstained with DAPI (Sigma). Images were taken with an Olympus IX51 inverted fluorescence microscope and analyzed with Fiji [55]. For each individual sample, n = 10 fields of view were manually counted. Ratios of PCNA, γH2AX or cleaved Caspase-3 positive cells were determined using Prism 9.1.2. *p*-values below 0.05 were considered significant. Statistical tests and samples sizes as indicated in the figure legends. 

### 4.5. Genomic PCR and Quantitative RT-PCR

The isolation of genomic DNA and RNA was performed with the QIAamp DNA Micro Kit (Qiagen, Hilden, Germany) or AllPrep DNA/RNA/Protein Micro Kit (Qiagen). For the conventional genomic PCR, 20 ng of gDNA was amplified. For the genomic qPCR, 50 pg of DNA was amplified on a LightCycler 480 (Roche, Basel, Switzerland) with iTaq universal SYBR green supermix (Bio Rad). For the analysis of mRNA expression, cDNA was synthesized from 10 ng RNA and an RT-PCR was performed on a CFX96 Connect Real-Time PCR Detection System (Bio Rad, Hercules, CA, USA) with SsoAdvanced universal SYBR green supermix (Bio Rad). For primers, see Supplemental Tables S1 and S2.

### 4.6. Western Blot Analysis

Protein isolation was performed with the AllPrep DNA/RNA/Protein Micro Kit (Qiagen), according to the manufacturer’s specifications. In short, at the end of the isolation procedure, total protein extracts recovered from the AllPrep protocol were precipitated in acetone overnight. The pellet was air-dried, and proteins were redissolved in sample buffer. The samples were boiled and loaded onto Mini-PROTEAN TGX precast gels (Biorad). Proteins were transferred onto a PVDF membrane (Trans-Blot Turbo Transfer System, Biorad) and probed with the following antibodies: Mad2l1 (1:1000, 4636, Cell Signaling); tdTomato (1:500, 600-401-379, Rockland, Limerick, PA, USA); and H4 (1:1000, 61521, Active motif, Carlsbad, CA, USA). HRP labelled Goat anti-Mouse or Rabbit secondary antibodies were used to visualize protein expression using chemiluminescence substrate (SuperSignal West Dura Extended Duration Substrate, Thermo Scientific, Waltham, MA, USA) on a ChemiDoc imaging system (Biorad). The analysis and densitometry of the protein bands was performed with ImageLab (V5.0) software.

### 4.7. Single Cell Whole Genome Sequencing

The pediatric medulloblastoma samples used for scWGS were obtained following surgical resection at diagnosis. Informed consent was given, and local ethics committee approval was granted for use of the patient material. The scWGS and analysis were performed as described previously [33,56,57]. Anonymized reads of the medulloblastoma samples are deposited at the European Nucleotide Archive/ENA (https://www.ebi.ac.uk/ena/browser/home, accessed on 24 July 2022), accession number PRJEB54865. Reads of the CGNP samples have accession number PRJEB54869.

## Data Availability

Anonymized scWGS reads of the medulloblastoma samples are deposited at the European Nucleotide Archive/ENA (https://www.ebi.ac.uk/ena/browser/home, accessed on 24 July 2022), accession number PRJEB54865. scWGS reads of the CGNP samples are deposited at the European Nucleotide Archive/ENA (https://www.ebi.ac.uk/ena/browser/home, accessed on 24 July 2022), accession number PRJEB54869. Raw data are available from Mendeley data, doi: 10.17632/sv4hjjgmkt.1 (https://data.mendeley.com/, accessed on 24 July 2022).

## Supplementary Materials

The following supporting information can be downloaded at: https://www.mdpi.com/article/10.3390/ijms23179852/s1.
