# Supplementary material for: Chromosomal Instability Characterizes Pediatric Medulloblastoma but Is Not Tolerated in the Developing Cerebellum
Source: Int J Mol Sci. 2022 Aug 30;23(17):9852. doi: 10.3390/ijms23179852 (PMC9456393; doi:10.3390/ijms23179852)

Supplemental information

Chromosomal instability characterizes pediatric medulloblastoma but is not tolerated in the developing cerebellum

Irena Bočkaj¶, Tosca E.I. Martini¶, Marlinde J. Smit, Inna Armandari, Bjorn Bakker, René Wardenaar, Tiny G.J. Meeuwsen-de Boer, Petra L. Bakker, Diana C.J. Spierings, Eelco W. Hoving, Victor Guryev, Floris Foijer, and S.W.M. Bruggeman.

Contents:

Supplemental figure S1: **Unsupervised hierarchical clustering analyses of dynamically expressed CGNP genes (of the yellow and light/dark blue gene groups) and orthologous human SHH medulloblastoma genes.**

Supplemental figure S2:
Allele switching efficiency and single cell sorting of CGNPs.

Supplemental table S1:
Primers for genomic PCR.

Supplemental table S2:
Primers for quantitative RT-PCR.

Uncropped images of Western blots and gels.

Figure S1

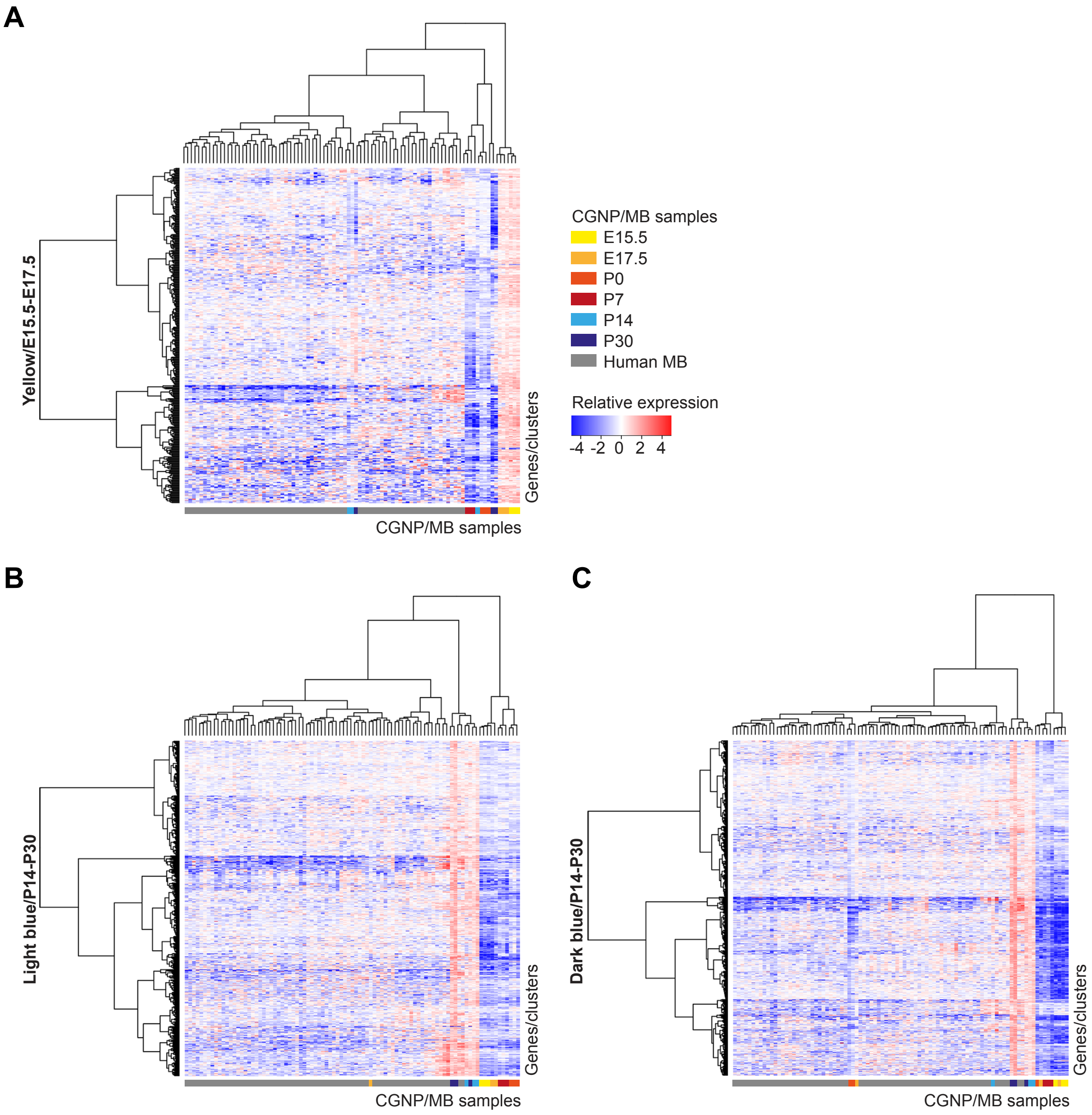

Supplemental figure S1:

Unsupervised hierarchical clustering analyses of dynamically expressed CGNP genes (of the yellow and light/dark blue gene groups) and orthologous human SHH medulloblastoma genes.

- **A.** Heatmap showing the cross-species unsupervised hierarchical clustering analysis of human SHH medulloblastoma genes and CGNP orthologous genes of the yellow/E15-E17 gene group (yellow CGNP genes as indicated in Figure 1D). Medulloblastoma and CGNP samples are plotted on the X-axis. Genes are plotted on the Y-axis.
- **B.** Heatmap showing the cross-species unsupervised hierarchical clustering analysis of human SHH medulloblastoma genes and CGNP orthologous genes of the light blue/P14-P30 gene group.
- **C.** Heatmap showing the cross-species unsupervised hierarchical clustering analysis of human SHH medulloblastoma genes and CGNP orthologous genes of the dark blue blue/P14-P30 gene group.

Figure S2

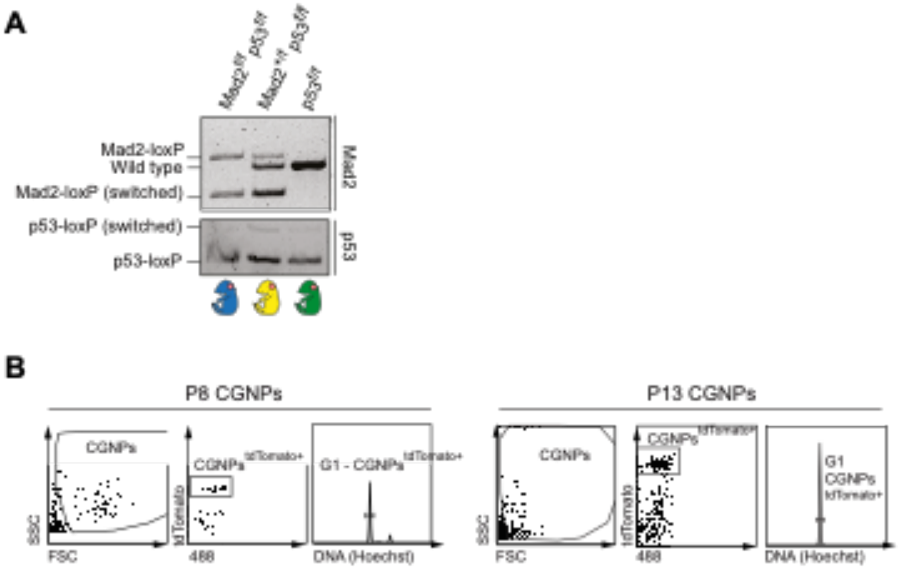

Supplemental figure S2:

Allele switching efficiency and single cell sorting of CGNPs.

A. Conventional genomic PCR assessing the switching efficiency of the floxed *Mad2l1* alleles in Mad2$^{f/f}$p53$^{f/f}$; Mad2$^{+/f}$p53$^{f/f}$ and p53$^{f/f}$ cerebella of postnatal day P7 mice. Upper panel: upper bands, floxed (unswitched) allele; middle bands, wild type *Mad2l1* allele; lower bands, switched *Mad2l1* allele. Lower panel: upper bands, switched *p53* allele; lower bands, floxed (unswitched) *Trp53* allele.
B. Representative FACS plots depicting the sorting strategy used to select for switched P8 (left panels) or P13 CGNPs (*i.e.*, tdTomato positive CGNPs)(right panels) in the G1 phase of their cell cycle. G1 CGNPs$^{tdTomato+}$ are directly sorted as single cells into 96 well plates for further processing. SSC, side scatter, FSC, forward scatter.

Supplemental table S1: **primers for genomic PCR**

**Conventional genomic PCR**	
**Primer name**	**Sequence**
**Mad2 5'**	AGGCTGAGCCGGGCCTTAGGAC
**Mad2 3' short**	CCCAGTTGAGAATGACATTTGAGAAGG
**Mad2 3' end of gene**	GCAGACCAAACGAACCTAAGTT
**P53 delta optimized del PCR RV**	AAGGCTTGGAAGGCTCTAGG
**P53 optimized del PCR RV**	GGAGGCAGAGACAGTTGGAG
**P53 intron 2 FW corrected**	GCAAACATGATACCCTTGGT
**Quantitative genomic PCR**	
**Primer name**	**Sequence**
**gen 5p Qmad2 A F**	TTAGGGAGGGATTCGGAGTT
**gen 5p Qmad2 A R**	CAGGCGTAATGAGCCCTAAG
**gen del Qmad2 A F**	GTGACTGGCGGTGGTTAGAT
**gen del Qmad2 A R**	CAGAGCATCAGAACCGTGAA

Supplemental table S2: **primers for quantitative RT-PCR**

**q RTPCR**	
**Primer name**	**Sequence**
**Trp53 A Fw**	TGTTATGTGCACGTACTCTCC
**Trp53 A Rev**	GTCATGTGCTGTGACTTCTTG
**Trp53 B Fw**	TCCGAAGACTGGATGACTG
**Trp53 B Rev**	AGATCGTCCATGCAGTGAG
**Mad2l1 Fw**	AAACTGGTGGTGGTCATCTC
**Mad2l1 Rev**	TTCTCTACGAACACCTTCCTC

PCR gels (Figure S2A)

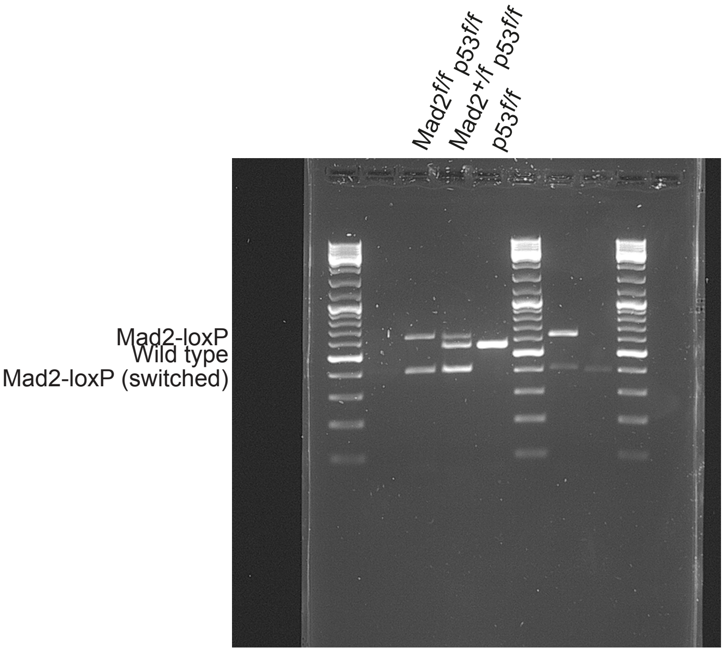

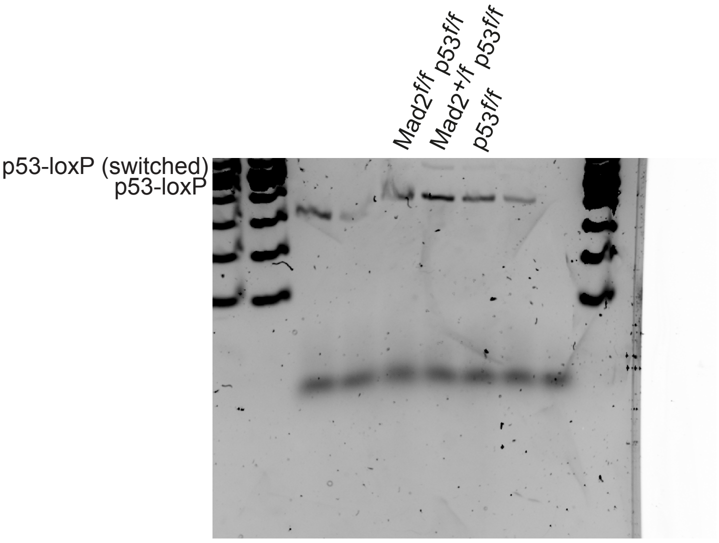

Supplement: Supplementary file 1 [file ijms-23-09852-s001.zip › ijms-1858160-supplementary.pdf]
